# Impact of Network Activity on the Spread of Infectious Diseases through the German Pig Trade Network

**DOI:** 10.3389/fvets.2016.00048

**Published:** 2016-06-21

**Authors:** Karin Lebl, Hartmut H. K. Lentz, Beate Pinior, Thomas Selhorst

**Affiliations:** ^1^Institute of Epidemiology, Friedrich-Loeffler-Institute, Greifswald, Insel Riems, Germany; ^2^Institute for Veterinary Public Health, University of Veterinary Medicine Vienna, Vienna, Austria; ^3^Unit Epidemiology, Statistics and Mathematical Modelling, Federal Institute for Risk Assessment, Berlin, Germany

**Keywords:** network analysis, disease spread, trade activities, temporal network, animal movements, epidemiology

## Abstract

The trade of livestock is an important and growing economic sector, but it is also a major factor in the spread of diseases. The spreading of diseases in a trade network is likely to be influenced by how often existing trade connections are active. The activity α is defined as the mean frequency of occurrences of existing trade links, thus 0 < α ≤ 1. The observed German pig trade network had an activity of α = 0.11, thus each existing trade connection between two farms was, on average, active at about 10% of the time during the observation period 2008–2009. The aim of this study is to analyze how changes in the *activity* level of the German pig trade network influence the probability of disease outbreaks, size, and duration of epidemics for different disease transmission probabilities. Thus, we want to investigate the question, whether it makes a difference for a hypothetical spread of an animal disease to transport many animals at the same time or few animals at many times. A SIR model was used to simulate the spread of a disease within the German pig trade network. Our results show that for transmission probabilities <1, the outbreak probability increases in the case of a decreased frequency of animal transports, peaking range of α from 0.05 to 0.1. However, for the final outbreak size, we find that a threshold exists such that finite outbreaks occur only above a critical value of α, which is ~0.1, and therefore in proximity of the observed activity level. Thus, although the outbreak probability increased when decreasing α, these outbreaks affect only a small number of farms. The duration of the epidemic peaks at an activity level in the range of α = 0.2–0.3. Additionally, the results of our simulations show that even small changes in the activity level of the German pig trade network would have dramatic effects on outbreak probability, outbreak size, and epidemic duration. Thus, we can conclude and recommend that the network activity is an important aspect, which should be taken into account when modeling the spread of diseases within trade networks.

## Introduction

Live animal trade represents an important economic sector but is permanently subject to fluctuations. For instance, consignments of pigs increased to 48% within EU-27 member states between 2005 and 2009 (1). However, the financial crisis in the subsequent years might have lessened this effect. The importance of live animal trade on the economy is also demonstrated during animal disease outbreaks. Trade restrictions with movement bans cause enormous financial losses for the affected livestock holdings and countries. For example, the outbreak of classical swine fever (CSF) in the 1990s in Germany led to an economical loss of approximately €1 billion (2). Thus, as demonstrated during CSF outbreak in Germany, livestock trade between farms is one of the major routes for the spread of animal diseases, although other infection routes, like proximity to infected herds or contact with contaminated persons and vehicles, exist as well (2).

Scientific research has primarily focused on the influence of the trade structure of farms on disease dynamics (3, 4). Farms differ with respect to their trade activity, i.e., with respect to the number of trading partners, trade connections, trade volume, and time intervals (5). Within the trade network, farms with greater trade activities are the most important contributors to disease spread (6). Veterinary epidemiology assessments utilized social network analysis (SNA) tools, such as centrality measures, developed within the field of social sciences, to calculate the importance of farms for the spread of animal infections. Numerous centrality measures, such as in- and out-degree, betweenness, and closeness (7), were correlated with standard epidemiological parameters, such as size of an epidemic, duration of the epidemic, time to peak of the epidemic, and the basic reproduction number *R*_0_ (4, 8–10).

Previous studies applying SNA on pig trade networks have already provided important insight for disease prevention and control. One aspect of this research was the identification of the structure of trade communities (11, 12). Another essential finding was that there is a large degree of heterogeneity associated with movements of pigs at the movement level and at the premise specific network level as well (13). As a result, pig trade has a right-skewed distribution of all centrality parameters, i.e., few holdings have high centrality, while most have a low centrality. Thus, strategic removal of the most central nodes would result in a decomposition of the network into fragments, which would interrupt infection chains and prevent further disease spread (14–16). It was also shown that the holding types differ in their centrality measures, which allow for a targeted removal of specific holding types in the case of a disease outbreak (16–18). Further, SNA has been utilized to simulate the spread of specific diseases to estimate the effects of an outbreak, e.g., the spread of Methicillin-resistant *Staphylococcus aureus* (MRSA) through the Danish pig trade network (19).

Although SNA provides useful insights into epidemic dynamics on trade systems, the methods used in SNA do not take into account the temporal ordering of trade links. Whenever a network is traversed using trade links, each traversal has to follow a causal sequence of connections. This constraint can have a significant impact on the spreading paths for pathogens in networks (20). For this reason, recent work has been focused on *temporal network analysis*, where each connection has a time stamp marking its occurrence time. The probability of contagion between two individuals is not constant in time and depends, beside the transmission rate and infectious period, also on the frequency and duration of the contact (21–24). Studies that considered the heterogeneity and duration of contacts and their importance for the epidemic showed the importance to elucidate the time dependency of activities in order to investigate disease dynamics (22, 24–26). Previously, it has been shown that the aggregation of trade links into static networks leads to an overestimation of the epidemic size (27–30), the outbreak probability (31), and the epidemic duration. Thus, scientific research in the veterinary field has increasingly focused on time-dependent networks. Methods have been adapted and extended from static analyses to time-dependent analyses (20, 27, 31–37).

A temporal network view on livestock trade networks includes the frequency of trade links. For the whole system, this frequency can be considered as the pace of trading. This raises the question, whether it makes a difference for a potential spread of an animal disease to transport many animals at the same time (low frequency) or few animals at many times (high frequency). From the economic point of view, it is appropriate to choose a low trade frequency and transport many animals at the same time.

In this work, we analyze the impact of the overall trade frequency on the spread of infectious disease. Hereby, we keep the total trade volume of the network constant and systematically investigate the impact of a changing frequency of traded animals. We define the *activity* of a network by averaging the frequency of all existing trade connections between node pairs and analyze how changes in the activity influence the probability of a disease outbreak, the final outbreak size, and the duration of an epidemic. A discrete stochastic SIR model is used to simulate the spread of a hypothetical disease through the trade network of the German pig production chain.

## Materials and Methods

In order to analyze the influence of network activity on the course of an epidemic, an outbreak model predicting the course of a hypothetical animal disease on a contact network between holdings belonging to the German pig production chain was set up. Besides the outbreak model, we propose a method how to systematically adjust the activity of the network.

### Data and Network Setup

According to the EU directive EC/2000/15 (38), EU member states are obliged to collect and record livestock movement data in a national database. Pursuant to the German Animal Movement Directive (Viehverkehrsverordnung), each holding in the pig production chain (including piglet production, breeding, raising, fattening, slaughtering, and trading) is obliged to notify the movement of pigs within 7 days. All data are stored in a database, “Herkunftssicherungs- und Informationssystem für Tiere” (HI-Tier). In Germany, movement data for pigs are collected on a daily basis. In general, movement data of livestock comprise information about the source and target farms (unique identifiers), the date of movement, and the number of animals moved (batch size).

For this study, pig movement data from the federal states of Bavaria and Baden-Württemberg between the years 2008 and 2009 were used. It has previously been shown that a period of 2 years is suitable to cover all characteristic properties of the German pig trade network (31). In our data set in most cases (90%), only one movement per week took place between a supplier and buyer. Consequently, we decided to use a weekly timescale for our analysis. In the case of two movements per week, those were merged into one occasion.

**Figure 1:**
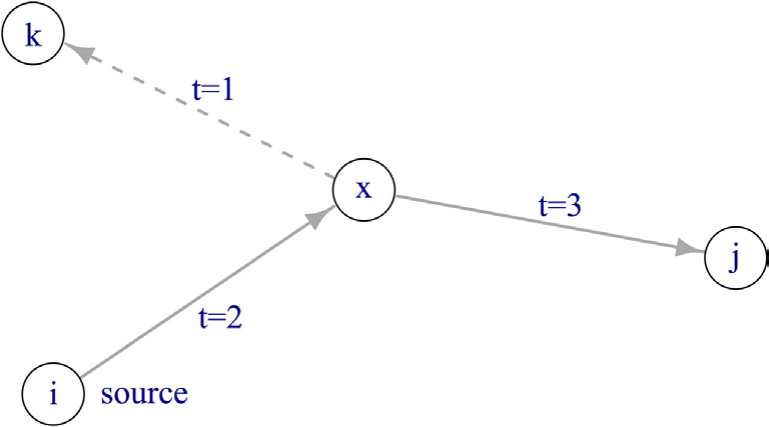
**Disease spread in a temporal network**. If the source node *i* is infectious at time *t* = 0, the disease can spread *via* node *x* to node *j*. Node *k* cannot become infected, as the edge between node *x* and *k* is active at *t* = 1, thus before the disease has reached node *x* at *t* = 2.

To describe the pattern of trade activity over time, a temporal network was constructed. By implementing a temporal network, it is possible to take into account causality for network transversal. In other words, consecutive trade connections have to be temporally ordered in order to make up a valid indirect connection between farms (Figure 1). The network comprised nodes and edges, where each edge connected a node pair. Farms were represented by nodes, and movements of animals between farms at a certain point in time were represented by directed edges. A temporal network is defined as 𝒢(*V*, ℰ, *T*), where *V* is the set of nodes within the network, ℰ is the set of directed edges, and *T* represents the length of the observation period, as we considered weekly time steps, *T* = 104 weeks. An edge (*u, v, t, w*)∈ℰ describes the movement of *w* pigs from farm *u* to farm *v* at time *t* ≤ *T*. This network comprises |V| = 45,065 and |ℰ| = 1,237,753 edges (i.e., overall number of transports during the observation period). Further, the static representation of the network was constructed by summing all observations in the temporal network over the study period, such as the static network is the time-aggregated network of 𝒢. In the static representation of the network *G*(*V, E*), *V* represents the set of nodes and *E* the set of directed edges (|*E*| = 112,826). A directed edge between two nodes exists in the static network if a certain animal movement has taken place at least once during the observation period.

The aim of this analysis was to investigate the influence of the network activity on the outbreak size of an epidemic. However, this outbreak size would be strongly influenced by differences in the reachability of the nodes, i.e., nodes form distinct reachability classes where a significant number of nodes may only cause trivial outbreak (11, 12). To reduce this bias, the data were first tailored to include only nodes, which are, in the static representation of the network, reachable from each other. We used the static network to identify the *largest strongly connected component* (LSCC; in a strongly connected component, each node is reachable by any other node in the component). The further analysis was limited to this LSCC, which we denote as G_*_. Thus, the static representation of the network enables the disease to reach all nodes in finite time, no matter which node is the source of infection. All nodes and edges, which were not elements of the LSCC in the static network, were removed, as well as the corresponding elements from the temporal network. We hereby implicitly assumed that the concept of connectivity (35, 36) is preserved for the temporal network. In the resulting network, pigs moved between |*V*| = 7,455 farms (number of nodes in the LSCC) and |*E*| = 27,149 transport routes (number of edges in the LSCC) were recorded during the observational period, corresponding to |ℰ| = 315,481 transports in the temporal network.

### Setting the Network Activity α

Starting from the network generated as described above, we changed the activity systematically. The *activity of a single edge* in a temporal network 𝒢 can be described by its frequency, i.e., how often a certain edge was active during the study period divided by the length of the study period. The *network activity* α was defined as the mean edge frequency of a network, with 0 < α ≤ 1. The network activity α of a temporal network 𝒢(*V*, ℰ, *T*) and its according static representation *G*(*V, E*) can be calculated as follows.

(1) $$\alpha =\frac{\left|ℰ\right|}{\left|E\right|\times T},$$

where |ℰ| is the number of edges in the temporal network, |*E*| is the number of edges in the aggregated network, and *T* is the observation period.

In order to investigate the influence of α on disease dynamics, we propose a method to systematically change the network activity. Since the results for a network with shifted α should be comparable to the original network, following constraints had to be considered: (i) the aggregated network *G* remained the same for all α, (ii) the total trade volume remained constant for all α, (iii) the temporal sequence of existing trade routes had to be preserved (see details below), and (iv) the observation period *T* was preserved.

In order to highlight the activity of a temporal network, we computed the activity according to Eq. 1 and denoted a temporal network with a certain network activity as 𝒢_α_. For our observed network, we found α = 0.11, and we denote the observed network as 𝒢_α=0.11_≡𝒢_*_.

In order to create networks with a reduced α, randomly chosen edges from 𝒢_*_(*V*, ℰ, *T*) were removed. According to constraint (i), edges were removed in a way such that each edge of the aggregated network appeared at least once in the newly generated temporal network.

In order to increase α, we first considered our temporal network as a sequence of static network snapshots. In other words, a temporal network consists of an ordered sequence 𝒢_α_(*V*, ℰ, *T*) = *G*_1_, *G*_2_, … , *G*_T_, where each *G*_t_ ∈ 𝒢 is a static snapshot of the temporal network at time *t*. In order to increase α, each snapshot was first duplicated (once or multiple times) and time-shifted by a certain value chosen at random. Second, these snapshots were merged into a new temporal network. In the case of overlapping edges occurring between the same node pairs (i.e., multiple occurrences of directed edges active at the same time; regardless of their edge weights), the edge weights *w* (i.e., number of transported pigs) were averaged. Using this approach, the existing trade routes remain preserved as required by constraint (iii).

In order to satisfy constraint (iv), we used periodic boundary conditions, i.e., for each edge *(u, v, t, w)* = (*u, v, t* + *T, w*). In other words, if the new times exceeded the observation period *T*, the times were shifted by subtracting *T*.

The procedures described above would already be sufficient to change the activity α of the observed network 𝒢_*_. Nevertheless, both procedures would violate constraint (ii), as the overall sum of edge weights changes as well. Therefore, the new edge weights had to be adjusted. During the observation period, a total of *W* = 24,995,162 transported pigs were recorded. The new edge weights for 𝒢_α_ were normalized, so that the sum of the new edge weights equaled the total of the observed edge weights *W*. Finally, edge weights for the generated network were rounded to a whole number, with the minimum number of pigs per transport set to one [constraint (i)].

**Figure 2:**
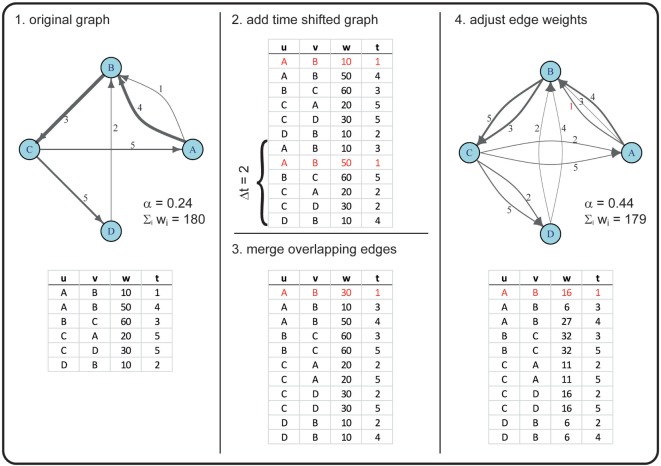
**Example for creating a graph with an increased α using a time shift of two time steps**. In this example, the original graph has |*V*| = 4 nodes (A, B, C, D) and |ℰ| = 6 directed edges (with *u* as the starting node and *v* as the receiving node), corresponding to |*E*| = 5 in the time-aggregated network. The edges are active at times *t* ϵ {1, 2, … , 5} (numbers next to the drawn edges), thus *T* = 5. The line widths of the edges correspond to the edge weights *w*. Overlapping edges (i.e., edges with the same *u, v, t*) are marked in red. The newly generated graph has the same number of nodes, but an increased number of edges (|ℰ| = 11). Note that, due to rounding errors, the sum of edge weights in the original and the new graph are only approximately equal.

For example, in a first step, we duplicated the graph 𝒢_*_ once and conducted a 52-week shift (i.e., a shift of 1 year in the duplicate). Thus, an edge active in the original graph at weeks 2, 40, 63, and 92 would be active in a 52-week-shifted graph at weeks 54, 92, 11, and 40. Merging the original with the time-shifted graph would thus result in a graph where this certain edge is active at weeks 2, 11, 40, 54, 63, and 92 (see Figure 2 for a more detailed example).

Overall, 22 different networks were generated with different activity values, including the original network 𝒢_*_ = 𝒢_0.11_. The considered values for α were approximately evenly distributed in the interval (0; 1].

### Disease Dynamics

#### SIR Model

In order to analyze the influence of network activity α on the course of an epidemic, we simulated the spread of a disease on different temporal networks 𝒢_α_ with parameter α. Disease dynamics were modeled by applying a stochastic discrete-time SIR model (29). Farms were treated as epidemiological units that are assigned to one of the three epidemiological states: susceptible (*S*), infected (*I*), and recovered (*R*). The infection spread along an edge (*u, v, t, w*), if at time *t* the supplying node *u* was in state *I*, and the receiving node *v* in state *S*. Thus, a receiving farm could only became infected, if a transport took place from the supplying farm to the receiving farm during the time period in which the supplying farm was in the *I* state. Infectious nodes stayed in the *I* state for μ time steps, thereafter they passed to the *R* state. Nodes in the *R* state remained in this state until the end of a simulation run. Infectious farms infect susceptible farms with probability *p_e_*.

Due to the fact that certain information was not available, the following model assumptions were made. (I) farms representing the nodes within the network were all treated identically (8, 29, 31, 39). Thus, in this model, the number of animals on the farm, breed, farm type, or farm practices did not have an effect on the transmission dynamics. (II) the epidemiological status does not alter the trade contact structure. The latter is a strong assumption, but it allowed an examination of the influence of network topology on unmanaged disease dynamics (29).

#### Model Parameters

In order to compute the transmission probability *p_e_* for each edge, we first considered the risk of infection for each transported animal. For every transport from an infected to a susceptible farm, each transported animal has a probability *p* to infect the receiving node. In this work, the probabilities *p* = {0.25, 0.5, 0.75, 1} were considered. The receiving node became infected, when at least one transported animal spread the disease. The probability *p_e_* can be described with a binomial function *B*(*w, p*), whereas the function depends on the parameters edge weight *w* and an animals’ transmission probability *p*.

(2) $$p_{e}=P\left(X>0\right)\sim B\left(w,p\right)=1-{\left(1-p\right)}^{w}.$$

A transmission probability of *p* = 1 corresponds to a highly infectious disease: the supplied farm always became infected, independent of the batch size *w*. This corresponds to a worst-case scenario and is therefore often used in studies investigating the spread of diseases within the trade networks (6, 8, 31).

Nodes remain in the *I* state for the *infectious period* μ and then pass to the *R* state. Nodes in the *R* state remained in this state. In this paper, we considered a constant infectious period of μ = 4 weeks [as estimated for cases, such as CSF, African swine fever, foot-and-mouth disease; Ref. (40)].

#### Initial Conditions

In the analysis presented here, the model predicted the disease dynamics for discrete intervals of 1 week. Initially, all farms were in the susceptible state (*S*). At a randomly chosen time, the state of one randomly selected farm was set to infected (*I*).

The disease dynamics were simulated on a temporal network 𝒢_α_. All possible start times and initially infected nodes (index nodes) had the same selection probability. The start times were selected from the interval [1; *T* − 40] to avoid that the durations of the epidemics exceed the observation period of 104 weeks. We chose 40 weeks arbitrarily, as the first test runs showed that the duration of the epidemic only rarely exceeded this time period. However, in some cases, the duration of the epidemic still exceeded the study period – those cases were excluded from the further analysis. The simulation stopped when the number of infectious nodes reached 0.

#### Summary of Parameters

For each value of the activity parameter coming from one of the 22 investigated 𝒢_α_, each with the 4 transmission probabilities as described above, the simulation was repeated 2,000 times, as test runs showed that this number of iterations provided robust results. Thus, 176,000 simulation experiments were run in total (Table 1). In 175,877 of those simulations, the duration of the infection did not exceed the observation period and were used for further analysis.

**Table 1:** **Description and value bound of the used parameters**.

	Parameter	Description	Value
Network	α	Network activity	22 values in the interval (0; 1]
Infection parameters	*p*	Infection probability per transported animal	{0.25, 0.5, 0.75, 1}
	*p_e_*	Infection probability per edge, depending on batch size	calculated according to Eq. 2
	μ	Infectious period	4 weeks (constant)
Initial conditions	*u*	Starting node	2,000 random samples from *V*
	*t*	Starting time	2,000 random samples from [1; *T* − 40]
Total runs			176,000

### Analysis

We wanted to determine the probability that a disease outbreak occurs for a certain level of α. The *outbreak probability* was estimated as the proportion of the 2,000 simulation runs, in which the disease spread beyond the starting node. In those cases where the disease spread beyond the starting node, the *outbreak size* was calculated as the total number of infected nodes. In addition, the *outbreak duration* was defined as the number of weeks in which infected nodes occurred. The distribution of the latter two measures was skewed to the right, and thus we give the median and the first and third quartiles (Q1, Q3).

All analyses were conducted using the open-source software *R* version 3.2.1 (41). The package *igraph* (42) was used to generate and analyze the network.

## Results

### Descriptors of G_*_

For this static representation G_*_, we found an average shortest length of 6.33; the path length between the two most distant nodes (diameter) was 17. The median in-degree, measuring the number of trade partners delivering animals to a certain node was only one, while the median for the number of trade partners a certain holding delivers to (out-degree) was two (Table 2). The values for the median ingoing and outgoing closeness centrality were rather similar (Table 2), indicating that the number of steps required to reach a certain node equals the number of steps required to reach any other node from a certain node. The number of shortest paths going through a certain node (betweenness centrality, Table 2) showed a high variation, ranging from 0 to 15,166,160.

**Table 2:** Minimum, 25% quartile, median, 75% quartile and maximum of the calculated centrality parameters for G_*_, the static representation of the observed network.

	Min	Q1	Median	Q3	Max
In-degree	1	1	1	2	665
Out-degree	1	1	2	3	358
Ingoing closeness centrality	0.000011	0.000018	0.000023	0.000026	0.000041
Outgoing closeness centrality	0.000012	0.000019	0.000022	0.000024	0.000037
Betweenness centrality	0.0	1.0	316.2	7,490.2	15,166,160.0

### Outbreak Probability

**Figure 3:**
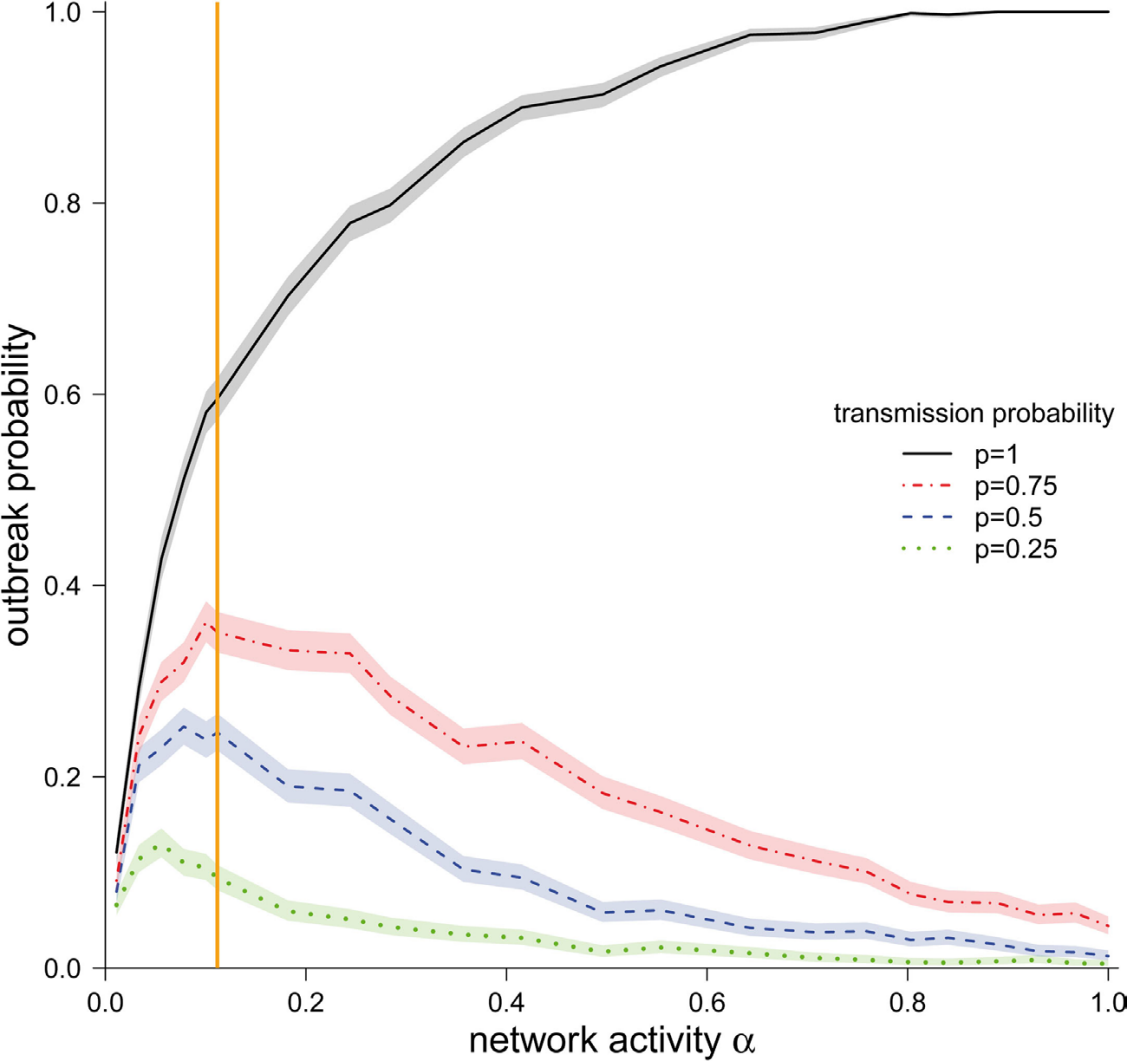
**Outbreak probability (±95% CI) depending on the network activity level α for different disease transmission probabilities *p***. The vertical orange line represents α for the observed pig trade network 𝒢_*_.

We observed that the outbreak probability is finite, independent of the particular values of transmission probability *p* and network activity α (Figure 3). Even for the smallest considered activity values (α = 0.01), the outbreak probabilities was in the region of 5% for all considered transmission probabilities.

We now focus on the outbreak probability for a transmission probability of *p* = 1, i.e., the worst-case scenario, in which transports of any size spread the infection. In this scenario, a monotonous increase of the outbreak probability with increasing activity was observed. The outbreak probability saturated for larger values of α. More precisely, the outbreak probability was greater than 99% for all α > 0.80. For small and intermediate values of α, it can be observed that even relatively small changes in α had a strong effect on the outbreak probability. Our observed network (α = 0.11) lies in this region. Consequently, small changes in the real system would result in large changes in the outbreak probability.

We now focus on transmission probabilities of *p* < 1. For all considered *p* < 1, a qualitatively similar behavior could be observed. Contrary to the worst-case scenario (*p* = 1), the outbreak probabilities for *p* < 1 did not increase monotonously, but rather showed a maximum. The location of these maxima was shifted to the right for increasing values of *p*. It should be noted that the location of these maxima was relatively close to the activity of the observed network 𝒢_*_.

### Final Outbreak Size

**Figure 4:**
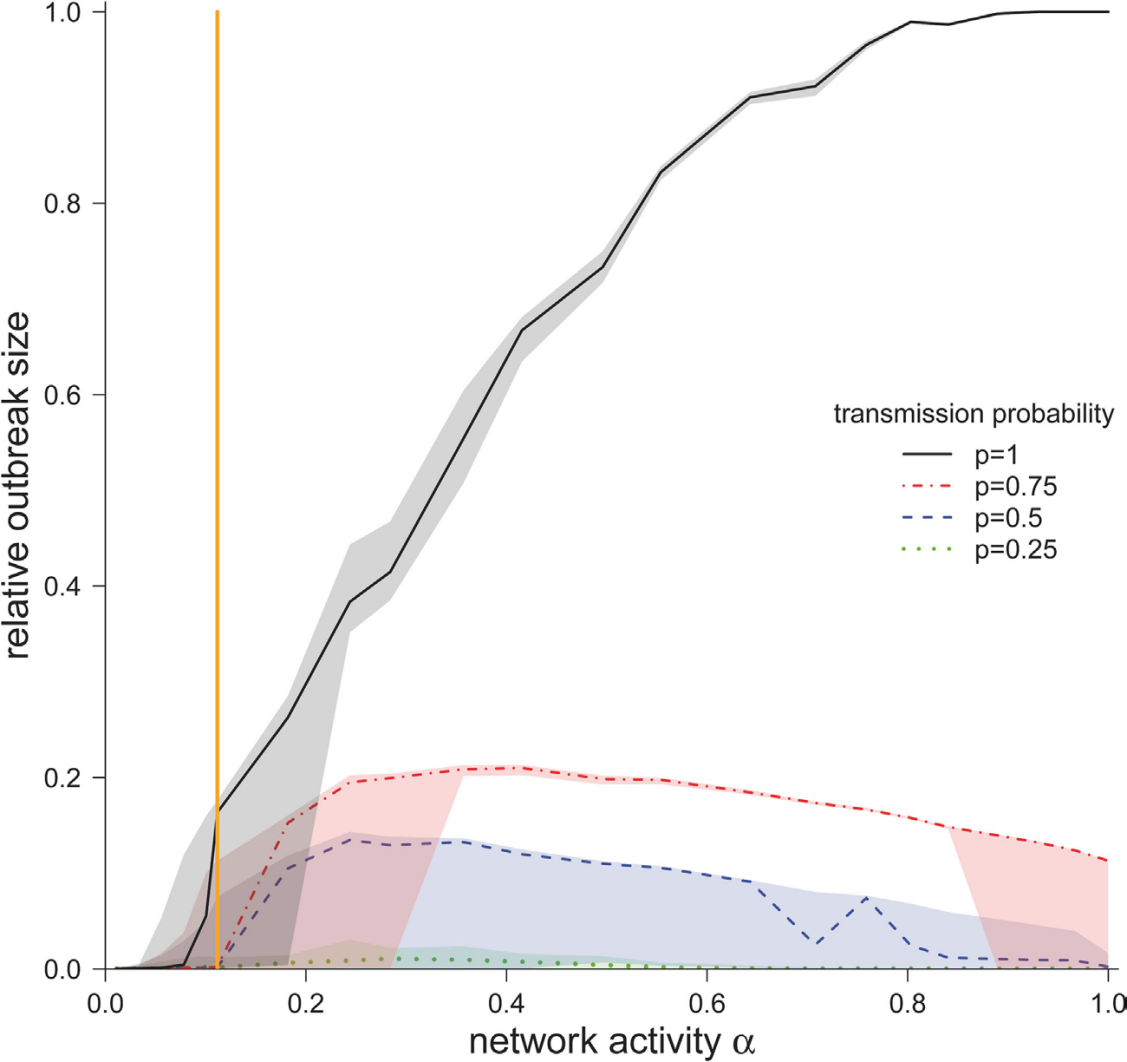
**Median outbreak size (measured as a fraction of total number of nodes) ± quartiles (Q1 and Q3) for different disease transmission probabilities *p* (in the case the disease spread beyond the starting node)**. The vertical orange line represents α for the observed pig trade network 𝒢_*_.

We now consider the cases where the infection spread beyond the starting node and the corresponding outbreak sizes for different values for α and *p* (Figure 4). For the worst-case scenario *p* = 1, the outbreak size increased monotonously with increasing α. The possibility that all nodes in the network became infected was only found at this scenario (*p* = 1), but only for very high network activities. For the observed network (α = 0.11), ~15% of the nodes would become infected in the worst-case scenario.

For smaller transmission probabilities (*p* < 1), we observed that outbreak sizes are significantly smaller than in the worst-case scenario. In contrast to the worst-case scenario, the outbreak sizes showed a maximum at approximately α = 0.3.

The authors would like to stress the fact that the outbreak size showed a critical threshold regarding the network activity. This means that there was a *critical activity* α_crit_, such that finite outbreaks occurred only if α > α_crit_. To estimate α_crit_, we calculated the central point between the last value of α below and the first value above the threshold. For transmission probability *p* = 1, we found α_crit_ = 0.1, and for transmission probabilities of 0.75, 0.5, 0.25, we found α_crit_ = 0.15. Interestingly, the activity of the observed network was close to the critical region. For *p* = 1, the activity of the observed network 𝒢_*_ was only slightly above the critical threshold, whereas for transmission probabilities *p* < 1, the observed network was subcritical. As it is typical for such critical regimes, small changes in the activity result in large changes in the outbreak size (Figure 4).

### Outbreak Duration

**Figure 5:**
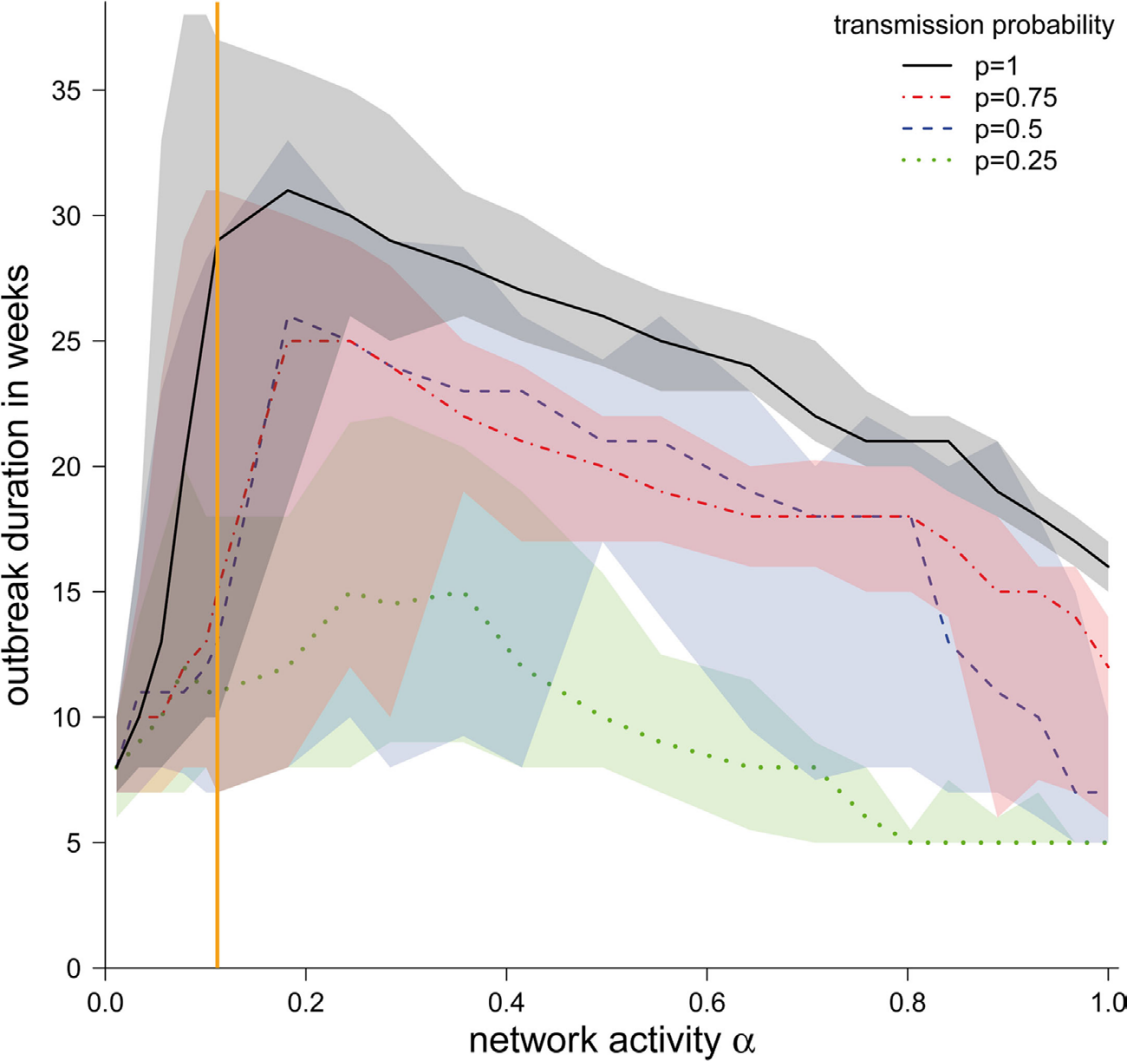
**Median duration of the epidemic in weeks (in the case the disease spread beyond the starting node) ± quartiles (Q1 and Q3) depending on the network activity level α for different disease transmission probabilities *p***. The vertical orange line represents α for the observed pig trade network 𝒢_*_.

Although the shapes of the outbreak durations were similar for different transmission probabilities, we found that the outbreak duration increased with higher transmission probabilities (Figure 5). However, for all transmission probabilities, a maximum in the outbreak probability at approximately α = 0.2 could be found, with the exception of *p* = 0.25, where the maximum was at approximately α = 0.3. The reason for these maxima is the existence of two dueling effects. (i) For small α, the outbreak duration correlates with the outbreak size. Outbreaks were typically small here, and increasing α increased the possible number of paths to other nodes. Topological and temporal shortcuts played a minor role here. (ii) For large values of α, the network was likely to form a number of shortcuts, accelerating the spread of a disease.

## Discussion

In this study, we investigated how the spreading of hypothetical infectious diseases through a trade network is influenced by the networks activity level. For the observed German pig trade network α = 0.11, thus each existing trade connection between two farms was on average active at about 10% of the time during the observation period (using weekly time steps). At this observed low network activity, the chances for a disease to spread beyond the starting node were relatively low, especially for low transmission probabilities (e.g., 10% at *p* = 0.25). Even in the case that an infection spread, the total number of infected farms was for all but the worst-case scenario only about 0.2% of the nodes within the network. Previously, the size of the largest connected component has often been used as an estimate for the potential final size of an epidemic spreading through a network (43). However, even at the applied worst-case scenario (*p* = 1), the size of the epidemic in our simulation was only a fraction (around 16%) of the total number of nodes for the observed trade data. Using the LSCC would therefore have considerably overestimated the final size of the epidemic. Thus, our results indicate that, at the observed level of the network activity, the threat of large epidemics spreading through the German pig trade network is relatively low, especially for diseases with low transmission rates. However, as we focused in our study on the spread of diseases through the trade network, the actual number of infected farms could be higher due to additional spreading *via* other infection routes (2).

For our analysis, we limited the trade network to the LSCC in order to avoid bias in the results caused by differences in the reachability of the nodes. The observed network activity of the untailored network (α = 0.105) was very similar to the activity in the LSCC (α = 0.112), indicating that changes from the observed network activity would have the same effect on the outbreak probability, -size, and -duration. However, due to the differences in the reachability of the nodes, a much higher variation in the results is to be expected (20).

Interestingly, the German pig trade network seems to be at a rather unstable state, as even small changes in the networks activity level would have a large impact on the spreading of diseases. The main factor that could change the network activity of the German pig trade network is likely to be the farm size. In the last years, the pig production in Germany and other EU countries increased, resulting in larger farm sizes and increased number of traded pigs (1, 44). This would also result in increasing animal transports, which could by archived either by increasing the animals per transport (i.e., edge weights) or by a higher frequency of transports (i.e., higher activity level), whereby the latter would likely have a higher impact on disease dynamics. If an increase in the network activity is to be expected in the long term, the probability for an outbreak and outbreak size are likely to increase, as shown in this study. Considering all three investigated measurements (outbreak probability, final outbreak size, and duration of epidemic), it becomes apparent that an increase in the network activity should be avoided. Further, in order to confine disease spreads, a decrease in the activity of the German pig trade network would be conducive, even if this reduction would only be minor. In our model, a decrease of the activity is realized by random deletion of edges. We assume that a targeted deletion of edges might even have a larger effect (45). From a practical point of view, a reduction in the network activity would mean that animal transports from one farm to another would have to be concentrated to fewer occasions. This also implies that a matching pig production schedule would be necessary, favoring “all-in-all-out” production systems.

The final outbreak size for different network activities shows, as depicted in Figure 4, strong similarities to the threshold behavior known from epidemic SIR-type models (46). This epidemic threshold describes a condition above which an epidemic becomes global, while below this threshold only a limited number of nodes become infected (46, 47). To estimate the epidemic threshold in a given network is thus important as it allows predicting the possibility that an infection spreads on a large scale. Hence, it is essential for the planning control and intervention strategies. Different methods exist to identify the epidemic thresholds, with the performance of those methods depending on the topology of the network (48, 49). The results of our study show not only the existence of a threshold but also that its position varies with the transmission probability. Read and colleagues (22) demonstrated for a small-scale human contact network that the encounter rate had a strong effect on the outbreak size at high transmission rates but could find no significant effect at low transmission rates. This concurs with our results, where the effects of the network activity on the outbreak size were most produced at high transmission rates. Again, it seems that the actual activity of the investigated system is close to this threshold value, as even a small increase in the activity level has a large impact on the outbreak size of an epidemic.

The outbreak probability peaked in a region below this threshold for a global epidemic. As the total number of transported animals was kept constant for all network activities, the batch sizes per transport increased, while the frequency of transports decreased at low network activities. Thus, as the edge infection probability *p_e_* depends on the batch size, the chance to transmit a disease beyond the starting node is rather high at low network activity levels, given the case that a transport occurs. As the number of transports is low at low levels of α, the epidemics are restricted to only a few livestock holdings. On the other hand, a decrease in the observed outbreak probabilities for large values of α can be observed, which can be explained by the fact that the batch sizes are small in this regime. Thus, disease spread was dominated by strong fluctuations in edge infection probability *p_e_*. These described effects only apply to transmission probabilities <1, as in the case of *p* = 1, the spreading of diseases is independent of the batch size.

As the necessary information was not available to us, we had to made several simplifications for our analysis. Especially, the farm type has already been shown to be an important factor in the spreading of the disease in animal trade networks (15). The farm type defines how long animals remain at a certain node. It is likely, that if the network activity would change due to an overall increase or decrease in the German pig production, the change in the activity of the individual trade connections would be irregular and vary according to the type of the source and the receiving node. This would be an important point to consider in further studies, as heterogeneous waiting times have been shown to influence the spread of diseases in networks (50, 51). For our simulation, we neglected within-herd transmission dynamics as well. Within-herd transmission depends not only on the specifics of a disease but is also influenced by several external factors that were not available to us (e.g., farm size or biosecurity measures on the farm level). The numbers of infected animals within a farm vary over time (52), and it is unlikely that all animals are simultaneously infected over a certain time period, as assumed in our simulation. Consequently, the presented results could overestimate the probability of a disease outbreak and the size of the epidemic. For our model, we assumed that the epidemiological status of the farms does not alter the trade contact structure. This applies to rather harmless diseases, like porcine reproductive and respiratory syndrome (PRRS), porcine circovirus type 2 (PCV2), or MRSA. However, depending on the severity of a disease, trade connections with an infected farm could cease. The withdrawal of trade connections would not be instantaneous but depend on various factors like incubation period or the occurring of clinical symptoms, resulting in a high variation between the time of infection of a farm and the potential termination of trade connections. Thus, the more likely a disease results in trade restrictions and the faster those restrictions are applied, the more our model is prone to overestimate the size of an epidemic. In case of an outbreak of a severe disease, trade connections could change due to the targeted implementation of trade restrictions by veterinary authorities. However, the extent of trade restrictions often differs between countries. For instance, during the bluetongue virus outbreak in Europe starting in 2006, trade restrictions in France were directed to specific areas (53), while in Germany, as well as in Austria and Swiss, the whole country was declared a single restriction zone at an early stage of the epidemic (54–56). Thus, if the whole country is declared a single restriction zone, the within-country trade network would likely show only marginal changes. The effect of lowering the contact rate on outbreak probability, -size, and -duration is shown in this analysis, but the implementation of trade restrictions directed to specific areas could lead to different dynamics.

In our study, we presented the *network activity* as a new indicator value for networks. With this parameter, it is possible to investigate how changes in the mean frequency in the activation of existing trade connections can affect the spread of diseases. By setting the total trade volume constant, as we did in this study, it was possible to differentiate between effects of trade frequency and trade volume. There are two specific characteristics of α: first, it is designed to be a characteristic of a temporal network. It has been shown that several network parameters drawn from a static network correlate with standard epidemiological parameters. Especially in networks with a right-skewed degree distribution, as we found for the pig trade network, nodes with a high degree can play an important role in the spreading of diseases (8, 14, 16). However, the frequency of trade links cannot be represented by a static network; static networks generated from different levels of α would be identical and thus network measurements (like centrality measurements) would be identical as well. As static networks do not take the temporal causality of the paths into account, results drawn from such static representations can be problematic. For example, it has been shown that compared to a temporal network, its static representation overestimates the size of a disease outbreak (20). Thus, in the last years, measurements for temporal networks have been developed (20, 57), and their relation to disease spread, however, remains to be investigated. In comparison with most of those measurements, the calculation of the *network activity* is simple, as it is obtained from the total number of edges in the static and the temporal network. Second, the *network activity* is a measurement for the state of the whole network and not for single nodes. It can be used as a measurement of how well a temporal network is described by its static representation. An α = 1 would be equal to network, where each existing trade link is active at all time steps, thus the static representation would be true at any time. The more closely the network activity is to one, the more accurate is its static representation. Still, for now, we would like to suggest carefulness in applying the results to other networks. While the general pattern is likely to stay the same, the exact location of the maxima/threshold of the investigated parameters could vary. Further, when comparing the *network activity* of different networks, care must be taken to use the same time period and time steps, as α changes with those two values.

In this study, we could demonstrate that the network activity α is an important factor in evaluating the effects of a disease spread in the German pig trade network. We would like to propose applying this indicator number to other networks used to demonstrate the spread of disease or other malicious agents as well, as the networks’ activity is likely to have a strong impact on the spreading.

## Author Contributions

This work was designed by TS, KL, and HL. BP, HL, and KL processed the raw data, and KL performed the data analysis. The results of the analysis were interpreted by KL, HL, and TS. KL, BP, and HL drafted and wrote the manuscript. All authors revised the manuscript and approved to the final version.

## Conflict of Interest Statement

The authors declare that the research was conducted in the absence of any commercial or financial relationships that could be construed as a potential conflict of interest.

## References

[1] Baltussen W Gebrensbet G deRoest K Study on the Impact of Regulation (EC) No 1/2005 on the Protection of Animals during Transport. (2011). Final Report. Specific Contract N SANCO/2010/D5/S12.574298.

[2] Fritzemeier J Teuffert J Greiser-Wilke I Staubach C Schlüter H Moennig V. Epidemiology of classical swine fever in Germany in the 1990s. Vet Microbiol (2000) 77:29–41. 10.1016/S0378-1135(00)00254-6 11042398

[3] Christley RM Pinchbeck GL Bowers RG Clancy D French N Bennett J Infection in social networks: using network analysis to identify high-risk individuals. Am J Epidemiol (2005) 162:1024–31. 10.1093/aje/kwi308 16177140

[4] Keeling M Eames K. Networks and epidemic models. J R Soc Interface (2005) 2:295–307. 10.1098/rsif.2005.0051 16849187 PMC1578276

[5] Pinior B Platz U Ahrens U Petersen B Conraths F Selhorst T The German Milky Way: trade structure of the milk industry and possible consequences of a food crisis. J Chain Netw Sci (2012) 12:25–39. 10.3920/JCNS2012.x001

[6] Pinior B Konschake M Platz U Thiele H Petersen B Conraths F The trade network in the dairy industry and its implication for the spread of contamination. J Dairy Sci (2012) 95:6351–61. 10.3168/jds.2012-5809 22999280

[7] Wasserman S Faust K Social Network Analysis – Methods and Applications. Cambridge: Cambridge University Press (1994).

[8] Natale F Giovannini A Savini L Palma D Possenti L Fiore G Network analysis of Italian cattle trade patterns and evaluation of risks for potential disease spread. Prev Vet Med (2009) 92:341–50. 10.1016/j.prevetmed.2009.08.026 19775765

[9] Bajardi P Barrat A Savini L Colizza V Optimizing surveillance for livestock disease spreading through animal movements. J R Soc Interface (2012) 9:2814–25. 10.1098/rsif.2012.0289 22728387 PMC3479905

[10] Lentz HHK Selhorst T Sokolov I. Spread of infectious diseases in directed and modular metapopulation networks. Phys Rev E Stat Nonlin Soft Matter Phys (2012) 85:066111. 10.1103/PhysRevE.85.066111 23005166

[11] Lentz HHK Konschake M Teske K Kasper M Rother B Carmanns R Trade communities and their spatial patterns in the German pork production network. Prev Vet Med (2011) 98(2–3):176–81. 10.1016/j.prevetmed.2010.10.011 21111498

[12] Lichoti JK Davies J Kitala PM Githigia SM Okoth E Maru Y Social network analysis provides insights into African swine fever epidemiology. Prev Vet Med (2016) 126:1–10. 10.1016/j.prevetmed.2016.01.019 26848113

[13] Bigras-Poulin M Barfod K Mortensen S Greiner M. Relationship of trade patterns of the Danish swine industry animal movements network to potential disease spread. Prev Vet Med (2007) 80:143–65. 10.1016/j.prevetmed.2007.02.004 17383759

[14] Nöremark M Håkansson N Lewerin SS Lindberg A Jonsson A. Network analysis of cattle and pig movements in Sweden: measures relevant for disease control and risk based surveillance. Prev Vet Med (2011) 99(2–4):78–90. 10.1016/j.prevetmed.2010.12.009 21288583

[15] Büttner K Krieter J Traulsen A Traulsen I. Efficient interruption of infection chains by targeted removal of central holdings in an animal trade network. PLoS One (2013) 8(9):e74292. 10.1371/journal.pone.0074292 24069293 PMC3771899

[16] Büttner K Krieter J Trraulsen A Traulsen I. Static network analysis of a pork supply chain in Northern Germany – characterisation of the potential spread of infectious diseases via animal movements. Prev Vet Med (2013) 110(3–4):418–28. 10.1016/j.prevetmed.2013.01.008 23462679

[17] Dorjee S Revie CW Poljak Z McNab WB Sanchez J. Network analysis of swine shipments in Ontario, Canada, to support disease spread modelling and risk-based disease management. Prev Vet Med (2013) 112(1–2):118–27. 10.1016/j.prevetmed.2013.06.008 23896577

[18] Rautureau S Dufour B Durand B. Structural vulnerability of the French swine industry trade network to the spread of infectious diseases. Animal (2012) 6(7):1152–62. 10.1017/S1751731111002631 23031477

[19] Ciccolini M Dahl J Chase-Topping ME Woolhouse ME. Disease transmission on fragmented contact networks: livestock-associated Methicillin-resistant *Staphylococcus aureus* in the Danish pig-industry. Epidemics (2012) 4(4):171–8. 10.1016/j.epidem.2012.09.001 23351369

[20] Lentz HHK Koher A Hövel P Gethmann J Sauter-Louis C Selhorst T Disease spread through animal movements: a static and temporal network analysis of pig trade in Germany. PLoS One (2016) 11(5):e0155196. 10.1371/journal.pone.0155196 27152712 PMC4859575

[21] Holme P. Epidemiologically optimal static networks from temporal network data. PLoS Comput Biol (2013) 9(7):e1003142. 10.1371/journal.pcbi.1003142 23874184 PMC3715509

[22] Read JM Eames KTD Edmunds WJ. Dynamic social networks and the implications for the spread of infectious disease. J R Soc Interface (2008) 5(26):1001–7. 10.1098/rsif.2008.0013 18319209 PMC2607433

[23] Stehlé J Voirin N Barrat A Cattuto C Colizza V Isella L Simulation of an SEIR infectious disease model on the dynamic contact network of conference attendees. BMC Med (2011) 9:87. 10.1186/1741-7015-9-87 21771290 PMC3162551

[24] Smieszek T. A mechanistic model of infection: why duration and intensity of contacts should be included in models of disease spread. Theor Biol Med Model (2009) 6:25. 10.1186/1742-4682-6-25 19919678 PMC2780993

[25] Colizza V Barrat A Barthélemy M Vespignani A. The role of the airline transportation network in the prediction and predictability of global epidemics. Proc Natl Acad Sci U S A (2006) 103(7):2015–20. 10.1073/pnas.0510525103 16461461 PMC1413717

[26] Chen S White BJ Sanderson MW Amrine DE Illany A Lanzas C. Highly dynamic animal contact network and implications on disease transmission. Sci Rep (2014) 4:4472. 10.1038/srep04472 24667241 PMC3966050

[27] Lentz HH Selhorst T Sokolov I. Unfolding accessibility provides a macroscopic approach to temporal networks. Phys Rev Lett (2013) 110:118701. 10.1103/PhysRevLett.110.118701 25166583

[28] Dubé C Ribble C Kelton D McNab B. Comparing network analysis measures to determine potential epidemic size of highly contagious exotic diseases in fragmented monthly networks of dairy cattle movements in Ontario, Canada. Transbound Emerg Dis (2008) 55:382–92. 10.1111/j.1865-1682.2008.01053.x 18840200

[29] Vernon M Keeling M Representing the UKs cattle herd as static and dynamic networks. Proc R Soc B (2009) 276:469–76. 10.1098/rspb.2008.1009 PMC2592553 18854300

[30] Karsai M Kivelä M Pan R Kaski K Kertész J Barabási AL Small but slow world: how network topology and burstiness slow down spreading. Phys Rev E Stat Nonlin Soft Matter Phys (2011) 83:025102. 10.1103/PhysRevE.83.025102 21405879

[31] Konschake M Lentz H Conraths F Hövel P Selhorst T. On the robustness of in- and out-components in a temporal network. PLoS One (2013) 8:e55223. 10.1371/journal.pone.0055223 23405124 PMC3566222

[32] Kao R Green D Johnson J Kiss I. Disease dynamics over very different time-scales: foot-and-mouth disease and scrapie on the network of livestock movements in the UK. J R Soc Interface (2007) 4:907–16. 10.1098/rsif.2007.1129 17698478 PMC1975769

[33] Casteigts A Flocchini P Quattrociocchi W Santoro N Time-varying graphs and dynamic networks. Int J Parallel Emergent Distrib Syst (2012) 27:387–408. 10.1080/17445760.2012.668546

[34] Holme P Saramäki J Temporal networks. Phys Rep (2012) 519:97–125. 10.1016/j.physrep.2012.03.001

[35] Kiss I Berthouze L Taylor TJ Simon P Modelling approaches for simple dynamic networks and applications to disease transmission models. Proc Roy Soc A (2012) 468:1332–55. 10.1098/rspa.2011.0349

[36] Nicosia V Tang J Musolesi M Russo G Mascolo C Latora V. Components in time-varying graphs. Chaos (2012) 22:023101. 10.1063/1.3697996 22757508

[37] Liu S-Y Baronchelli A Perra N Contagion dynamics in time-varying metapopulation networks. Phys Rev E Stat Nonlin Soft Matter Phys (2013) 87:032805 10.1103/PhysRevE.87.032805

[38] EUR-Lex. Directive 2000/15/EC of the European Parliament and the Council of 10 April 2000 amending Council Directive 64/432/EEC on health problems affecting intra-community trade in bovine animals and swine. Off J Eur Commun (2000) 43:34–5.

[39] Pinior B Conraths F Petersen B Selhorst T Decision support for risks managers in the case of deliberate food contamination: the dairy industry as an example. Omega Int J Manage S (2015) 53:41–8. 10.1016/j.omega.2014.09.011

[40] Fernandeź PJ White WR Atlas of Transboundary Animal Diseases. Paris: OIE (World Organisation for Animal Health) (2010). 277 p.

[41] Core Team R. R: A Language and Environment for Statistical Computing. Vienna: R Foundation for Statistical Computing (2015). Available from: http://www.R-project.org

[42] Csardi G Nepusz T The igraph software package for complex network research. Int J Complex Syst (2006) 1695 Available from: http://igraph.org

[43] Kao R Danon L Green D Kiss I. Demographic structure and pathogen dynamics on the network of livestock movements in Great Britain. Proc R Soc B (2006) 273:1999–2007. 10.1098/rspb.2006.3505 16846906 PMC1635475

[44] Roguet C Rieu M The German pork industry responds to societal demands: from private labels to sectoral initiative. Cahiers de l’IFIP (2014) 1(1):1–12.

[45] Borgatti S Identifying sets of key players in a social network. Comput Math Organ Theory (2006) 12:21–34. 10.1007/s10588-006-7084-x

[46] Pastor-Satorras R Castellano C Van Mieghem P Vespignani A Epidemic processes in complex networks. Rev Mod Phys (2015) 87:925 10.1103/RevModPhys.87.925

[47] Moreno Y Pastor-Satorras R Vespignani A Epidemic outbreaks in complex heterogeneous networks. Eur Phys J B (2002) 26(4):521–9. 10.1140/epjb/e20020122

[48] Shu P Wang W Tang M Do Y. Numerical identification of epidemic thresholds for susceptible-infected-recovered model on finite-size networks. Chaos (2015) 25:063104. 10.1063/1.4922153 26117098 PMC7112466

[49] Wang W Liu QH Zhong LF Tang M Gao H Stanley HE Predicting the epidemic threshold of the susceptible-infected-recovered model. Sci Rep (2016) 6:24676 10.1038/srep24676 27091705 PMC4835734

[50] Delvenne J-C Lambiotte R Rocha LEC. Diffusion on networked systems is a question of time or structure. Nat Commun (2015) 6:7366. 10.1038/ncomms8366 26054307

[51] Yang GL Yang X Optimal epidemic spreading on complex networks with heterogeneous waiting time distribution. Physica A (2016) 447:386–91. 10.1016/j.physa.2015.12.033

[52] Chis Ster I Dodd PJ Ferguson NM. Within-farm transmission dynamics of foot and mouth disease as revealed by the 2001 epidemic in Great Britain. Epidemics (2012) 4(3):158–69. 10.1016/j.epidem.2012.07.002 22939313

[53] Tago D Hammitt JK Thomas A Raboisson D. Cost assessment of the movement restriction policy in France during the 2006 bluetongue virus episode (BTV-8). Prev Vet Med (2014) 117(3–4):577–89. 10.1016/j.prevetmed.2014.10.010 25458706

[54] Gethmann J Hoffmann B Probst C Beer M Conraths FJ Mettenleiter TC Drei Jahre Blauzungenkrankheit Serotyp 8 in Deutschland. Tierärztl Umschau (2010) 65:4–12.

[55] Häsler B Howe KS Di Labio E Schwermer H Stärk KDC. Economic evaluation of the surveillance and intervention programme for bluetongue virus serotype 8 in Switzerland. Prev Vet Med (2012) 103(2):93–111. 10.1016/j.prevetmed.2011.09.013 22018548

[56] Pinior B Lebl K Firth C Rubel F Fuchs R Stockreiter S Cost analysis of Bluetongue virus serotype 8 (BTV-8) surveillance and vaccination programmes in Austria between 2005–2013. Vet J (2015) 206(2):154–60. 10.1016/j.tvjl.2015.07.032 26371833

[57] Nicosia V Tang J Mascolo C Musolesi M Russo G Latora V Graph metrics for temporal networks. In: Holme P Saramäki J, editors. Temporal Networks. Berlin, Heidelberg: Springer (2013). p. 15–40.

